# Emerging Roles of Noncoding RNAs in Bovine Mastitis Diseases

**DOI:** 10.3390/pathogens11091009

**Published:** 2022-09-03

**Authors:** Favour Oluwapelumi Oyelami, Tahir Usman, Prashanth Suravajhala, Nawab Ali, Duy N. Do

**Affiliations:** 1 The John Curtin School of Medical Research, Australian National University, Canberra, ACT 2601, Australia; 2 College of Veterinary Sciences & Animal Husbandry, Abdul Wali Khan University, Mardan 23200, KP, Pakistan; 3 Amrita School of Biotechnology, Amrita Vishwa Vidyapeetham, Clappana 690525, Kerala, India; 4 Department of Zoology, Abdul Wali Khan University, Mardan 23200, KP, Pakistan; 5 Faculty of Veterinary Medicine, Viet Nam National University of Agriculture, Hanoi 100000, Vietnam; 6 Department of Animal Science and Aquaculture, Dalhousie University, Truro, NS B2N 5E3, Canada

**Keywords:** miRNAs, circRNAs, lncRNAs, next generation of sequencing, mastitis

## Abstract

Non-coding RNAs (ncRNAs) are an abundant class of RNA with varying nucleotide lengths. They have been shown to have great potential in eutherians/human disease diagnosis and treatments and are now gaining more importance for the improvement of diseases in livestock. To date, thousands of ncRNAs have been discovered in the bovine genome and the continuous advancement in deep sequencing technologies and various bioinformatics tools has enabled the elucidation of their roles in bovine health. Among farm animals’ diseases, mastitis, a common inflammatory disease in cattle, has caused devastating economic losses to dairy farmers over the last few decades. Here, we summarize the biology of bovine mastitis and comprehensively discuss the roles of ncRNAs in different types of mastitis infection. Based on our findings and relevant literature, we highlighted various evidence of ncRNA roles in mastitis. Different approaches (in vivo versus in vitro) for exploring ncRNA roles in mastitis are emphasized. More particularly, the potential applications of emerging genome editing technologies, as well as integrated omics platforms for ncRNA studies and implications for mastitis are presented.

## 1. Introduction

**Figure 1:**
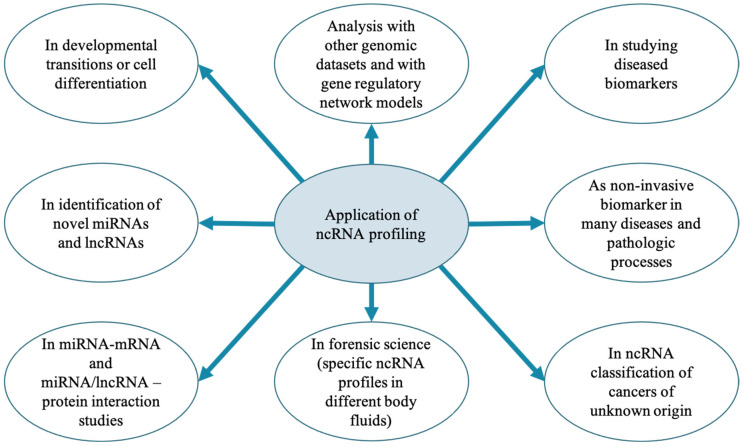
Potential roles of non-coding RNAs [19,20,21].

Non-coding RNAs include several groups of untranslated RNA molecules such as microRNA (miRNA), small interfering RNA (siRNA), small nucleolar RNA (snoRNA), PIWI-interacting RNA (piRNA), circular RNAs (circRNAs), and long non-coding RNA (lncRNA). They regulate gene expression and are involved in many biological processes. According to the ENCODE (ENCyclopedia of DNA elements) project results [1], a major portion of the genome is transcribed into noncoding regulatory elements or ncRNAs. The recently released miRbase v22 and NONCODE database (2017) contains about 1100 miRNAs and 22,386 identified lncRNA transcripts in the bovine genome. Interestingly, the ncRNAs have been shown to play an important role in multiple biological processes such as cell growth and development, differentiation, and metabolism, and have been used for various biological studies (Figure 1). The roles of ncRNAs in livestock species [2,3,4,5,6] and human diseases [7,8,9] have been comprehensively reviewed.

In dairy cows, the health of mammary glands (MG) is important for milk production and the health of the calves. However, the MG could be infected by various pathogens and inflammation (mastitis). Mastitis has a great impact on dairy production and has been intensively studied using omics approaches such as genomics [10,11,12], transcriptomics [13,14,15], and proteomics [16,17]. Growing evidence has shown that ncRNAs might play important roles in mastitis biology. A previous review on the roles of miRNAs in the mammary gland [2] had outlined important computational studies on miRNA in MG health and diseases. Moreover, Ibeagha-Awemu et al. [18] highlighted the roles of miRNAs and lncRNAs as important epigenetic markers for improving animal productivity and health. Later, Do et al. [4] covered recent studies on miRNAs and lncRNAs and discussed their diverse roles in ruminant studies. These reviews, however, lack information on the emerging aspects of ncRNA studies in mastitis. Therefore, given the importance of mastitis, in this review, we first discussed the biology of mastitis and its economic importance. We then provided updated state-of-the-art reports on the roles of various ncRNAs in mastitis and discussed the potential application of this knowledge for the genomic selection of dairy cattle against mastitis and how these important molecules could be harnessed for the development of important diagnostic and therapeutic molecular targets for mastitis.

## 2. Mastitis Biology

### 2.1. Concepts of Mastitis

**Figure 2:**
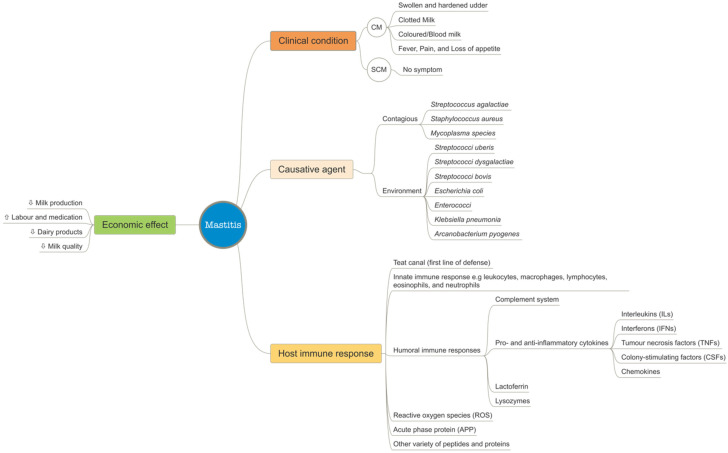
Some aspects of mastitis biology.

Bovine mastitis is an inflammation of the udder in lactating cows and is caused by a variety of pathogens, primarily bacteria [22]. This syndrome affects dairy animals’ health, and the quantity of milk produced, thus, causing a huge negative economic impact on the dairy industry [23,24]. The different aspects of mastitis biology and the subsequent effect on dairy production are shown in Figure 2.

### 2.2. Clinical Signs of Mastitis

Generally, there are two major forms of mastitis: clinical mastitis (CM) and subclinical mastitis (SCM), and several reports have shown that SCM is more prevalent than CM [13]. The CM is often characterized by a swollen and hardened udder, clotted milk, and sometimes blood in the milk, with the animal experiencing pain, fever, and loss of appetite. However, cows infected with SCM normally show no visible signs. Subsequently, milk obtained from cows with SCM is usually included in bulk milk, but that of CM is not [14].

### 2.3. Effects of Mastitis

Mastitis is considered an enemy of dairy farmers because of the loss in milk production, discarded milk in CM, increased labor, decreased dairy products due to low milk quality, premature culling, and increased medications and veterinarian services [25,26]. The costs for these factors might vary among countries and regions [27]. The estimated cost of mastitis is about EUR 80–180/cow/year in Western European production schemes [28], and in total, it causes an around USD 2 billion loss to the US dairy industry and an about USD 35 billion loss to the world dairy sector annually [29]. Generally, mastitis is known to cause changes in the physicochemical properties of milk as it could decrease the casein and lactose content of milk while increasing its whey (low-quality) protein content, consequently affecting the quality, flavor, and yield of dairy products such as cheese and lowering the amount of calcium in milk [23,30,31]. Additionally, this disease causes increased vascular permeability in the inflamed udder that, in turn, decreases the concentration of K+ and increases Na+ and Cl- in milk [32]. This increased vascular permeability also allows the passage of immunoglobulins, transferrin, serum albumin, and other serum proteins or metabolic features (associated with whey proteins) into milk. In general, mastitis-infected milk has the risk of bacterial contamination and antibiotic residues, thus, is unsuitable for human consumption. The effect of this is more prominent in developing countries where most milk produced by smallholder farmers is consumed unpasteurized, leading to a high rate of diarrheal disease among the population [33,34]. Even in the developed world where milk is usually pasteurized before consumption, some consumers still prefer unpasteurized products and are always at risk of developing foodborne diseases [35,36]. Apart from the effect of mastitis on milk quality, composition, and human health, it also greatly affects dairy processing and every stage of the production chain as reviewed by Garcia et al. [37]. This includes discarding contaminated trucks of milk and even attracts possible penalties for milk producers who are not meticulous with the processing stages.

### 2.4. Causative Agents of Mastitis

Bovine mastitis is caused by more than 100 pathogens including viruses, bacteria, fungi, and mycoplasma. The most frequent causative agents of mastitis are bacterial species such as Streptococcus, Staphylococci, and some Gram-negative bacterial species (Table 1). Based on causative agents, mastitis can be classified into two categories i.e., contagious mastitis and environmental mastitis.

The contagious mastitis is caused by *Staphylococcus aureus*, *Streptococcus agalactiae*, and *Mycoplasma* species. The contagious bacteria could spread from one teat to another, or from one cow to another cow of the same herd during milking. The chances of contagious mastitis can be reduced following appropriate hygienic measures [38]. Environmental mastitis accounts for about 1–10% of cases and is caused by common environmental germs such as different species of Streptococci (*uberis, dysgalactiae*, and *bovis*) and Coliforms (*Escherichia coli*, *Enterococci*, *Klebsiella pneumonia*, and *Trueperella pyogenes*) [38,39]. The most common source of these pathogens is fecal-contaminated bedding from where they gain entry into the teat canal of milking animals. The teat muscles act as a physical barrier by preventing the entry of pathogens into the canal. After milking, the teat canal remains open for a short time, which provides a chance for pathogens to gain entry into the teat canal of the animal while sitting on the contaminated ground after milking [38].

The impact of mastitis varies from mild to moderate infection depending upon the causative agent. *Staphylococcus aureus*, for example, has been reported as a cause of moderate but chronic infection. In this case, the infected animal must be removed from the herd because of the risk of spreading the bacteria in the herd [40]. On the other hand, Coliform species, i.e., *E. coli*, mostly cause severe acute inflammation in the udder [41]. Some strains of *E. coli* have been reported to cause more persistent conditions that may last for a longer time in some animals [42]. The prevalence of mastitis in several countries is shown in Table 1.

**Table 1:** Prevalence of cow-level and pathogen-wise mastitis in cattle.

Country	Cow Level Prevalence (%) of Mastitis			Pathogen-Wise Prevalence (%) of Mastitis					References
	CM ^1^	SCM ^2^	Overall Prevalence	*Staphylococcus* sp. ^3^	*Streptococcus* sp. ^4^	*E. coli*	*Klebsiella* sp.	Other ^5^	
**Argentina**	--	54	--	72.4	8.8	--	--	5.2	[43]
**Australia**	--	--	55	15	7.0	4	--	--	[44]
**Bangladesh**	--	51.0	51.0	45.7	14.8	9.9	--	30.9	[45]
**Canada**	--	--	36.2	20.6	--	2	--	2.9	[46]
**China**	--	--	--	39.0	11.0	--	--	18.2	[47]
**Kenya**	6.8	73.1	80.0	58.5	22.2	--	--	5.8	[48]
**Pakistan**	20	53	--	34.0	9.0	19.4	8	--	[49]
**Romania**	--	--	--	43.2	22.4	13.8	--	20.5	[50]
**Slovakia**	--	--	82.3	48.4	20.0	14.8	--	--	[51]
**Zimbabwe**	4.8	16.3	21.1	43.9	1.6	25.2	15.5	--	[52]

^1^ Clinical mastitis, ^2^ Subclinical mastitis, ^3^ *Staphylococcus* species recorded in the studies included *Staphylococcus* (CNS), *S. epidermidis*, and *S. Aureus*, ^4^ *Streptococcus* sp. recorded in the studies included *Str.uberis*, *Str. dysgalactiae*, and *Str. agalactiae*, ^5^ Other pathogens recorded in the studies in smaller percentages included *Proteus* sp., *Acinetobacter* sp., *Bacillus* sp., *Corynebacterium* sp., *Providentia* sp., *Aerococcus viridans*, *Pseudomonas,* and fungus.

### 2.5. Host Immune Responses to Mastitis

The defense mechanism in mammary glands depends on the complex coordination of nonspecific and specific protective elements, including the anatomical features of the mammary gland as well as cell-mediated and antibody-mediated immune components [53]. The smooth muscle sphincter, at the entrance of the teat canal, acts as a first physical barrier to prevent the entry of pathogens into the mammary gland. The stratified squamous epithelium of the teat canal act as a source of antimicrobial agents, such as keratin, that assist to combat the invading bacteria [54,55]. After the entry of the pathogen into the mammary gland, the mammary epithelium cells (MECs) are the first cells that encounter these pathogens. The MECs play a vital role in instigating the innate immune response by secreting inflammatory mediators, such as IL-8 which is a potent chemoattractant of neutrophils [56,57]. However, in certain conditions such as stress, drying-off, prepartum, loss of the keratin plug of the teat canal (that traps the invading pathogens), and post-milking, dilation of the sphincter muscles of the teat canal increases the invasion of pathogens and subsequent colonization that leads to intra-mammary infection (IMI) [32,54,58].

Innate immunity acts as a first line of defense in response to pathogens that penetrate the physical barriers of the teat canal [38]. It helps in pathogen recognition (PR) and pro-inflammatory responses to eliminate harmful pathogens. The innate immune response includes a wide variety of components, including different leukocytes, different components of humoral immune responses (i.e., complement system, pro- and anti-inflammatory cytokines, lactoferrin, and lysozymes), reactive oxygen species (ROS), acute-phase protein (APP), and variety of peptides and proteins. Most of these components are produced in other tissues of the body and are transported to the mammary gland [59,60].

Antimicrobial proteins and peptides (AMPs) are components of innate immune system that are expressed in early stages of bacterial infections and cause bacterial cell lysis [61]. The AMPs are secreted by epithelial cells at mucosal surfaces and in the skin. They also play an important role in intraphagosomal killing by neutrophils [62]. Different AMPs are identified including Cathelicidins, Defensin, Calcium-binding proteins, and lactoferrin that are expressed in epithelial cells of mammary gland and/or neutrophils [63,64,65]. Cathelicidins are one of the most important AMPs that play significant roles in innate and adaptive immunity. The Cathelicidins help in killing pathogenic microbes, perform immunomodulatory functions, and promote wound healing [66].

Neutrophils play a vital role in the second line of defense in the early stages of mammary gland infection. The neutrophils help in the phagocytosis of pathogens and the destruction of foreign materials. After performing their task, they undergo apoptosis and are removed by macrophages [67]. The number of neutrophils in milk SCC depends on the causative agent and the health status of the udder [68]. The inflammation of the mammary gland results in an increase in neutrophils and extracellular fluid that negatively affect the composition of milk [22]. Macrophages are another type of important immune cell that also contribute to eliminating pathogens by phagocytosis and their destruction by proteases and reactive oxygen species [69]. Natural killer (NK) cells, a subpopulation of lymphocytes, may play an important role in innate immune responses. These cells are cytotoxic in nature and are independent of the major histocompatibility complex (MHC). Cytokine-stimulated NK cells also have the ability to eliminate bacteria by secreting bactericidal proteins [29].

In the case of chronic infection, the innate immune response is not enough to eliminate the invading pathogens alone; thus, the humoral mechanism comes into action. In the humoral response, the T-helper cells release different cytokines in response to antigen recognition with MHC II. These cytokines stimulate B-lymphocytes that in turn produce a specific type of antibodies against the invading pathogens [58,70]. Of note, these immune-related molecules are coded for by specific genes which could be directly or indirectly regulated by various ncRNAs [15,71,72,73,74].

## 3. MiRNAs and Their Roles in Mastitis Biology

### 3.1. MiRNA Biosynthesis and Roles

Among the classes of short ncRNAs, the microRNAs have evolved to be one of the most abundant types. They are of varying lengths (about 22–23 nucleotides) and regulate the activity of about 60% of all protein-coding genes in mammals. They also control a variety of cellular processes by transcriptional or post-transcriptional repression of their target gene expression [75]. Generally, mature miRNAs are known to be produced in the cell through a cascade of biochemical events that are initiated in the cell nucleus and end in the cytoplasm [76,77,78]. These events occur in several key steps which can be briefly described as follows: First, the DiGeorge Syndrome Critical Region Gene 8 (DGCR8)/Drosha complex processes primary miRNA transcripts (pri-miRNAs) in the nucleus into precursor miRNAs (pre-miRNAs) which are subsequently processed into imperfectly paired miRNA duplexes by the dicer, in the cell cytoplasm. Finally, a single-stranded “guide” miRNA (from either strand of the matured miRNA) is preferentially incorporated onto the Argonaute (AGO) protein to form the RNA-induced silencing complex (RISC) [70]. Research has shown that most miRNA genes can be found in the introns of protein-coding genes and share the promoter with the host gene [79]. The miRNAs often have several transcriptional start sites. They regulate gene expression through induction of mRNA degradation, inhibition of either translation initiation or elongation processes, degradation of co-translational proteins, and premature termination of translation [75]. Additionally, they also can form miRNA–mRNA pairs which aid in informative biological mechanisms such as gene regulation. Since the research that led to the discovery of the first miRNA, *lin-4*, was published in 1993 [80], and with the advancement in deep sequencing technologies and developments of various bioinformatics tools for efficient processing of generated sequence data, several thousands of miRNAs have been discovered in humans, mice, farm animal species, and plants, and have been subsequently deposited in the miRNA database. This has indeed aided further understanding of the mechanism of miRNA regulation across species and cells. More importantly is that miRNAs are now being considered as potential biomarkers of several human diseases such as autoimmune diseases [81], metabolic disorders [82], and cardiovascular diseases [83]. The miRNA has also been shown to be a potential diagnostic marker and efficient therapeutic target in the diagnosis and treatment of various cancer types [84,85,86,87,88], majorly due to their crucial regulatory roles in many biological processes leading to these diseases.

### 3.2. Occurrence of miRNAs in MG Tissues

Abundant miRNAs have been identified in the bovine milk and tissues, supporting the identification of miRNAs in each tissue or disease condition as well as helping the development of a better non-invasive method of collecting tissue samples. Since Gu et al. [89] first identified 59 distinct bovine miRNAs in the bovine MG by cloning and sequencing extracted small RNAs from the MG tissue, the numbers of miRNAs identified in bovine tissues have increased sharply, thanks to the whole transcriptome shotgun sequencing (WTSS) methods. Moreover, there are about 38,589 miRNAs and as many as 93 clusters already reported for cattle in the latest version of miRBase (release 22, www.mirbase.org/ accessed on 25 August 2022 [90]). In the first miRNA study for bovine milk, Chen et al. [91] identified a total of 230 and 213 known miRNAs in the milk of 7 days postpartum and 90 days postpartum cows, respectively. Two years later, Izumi et al. [92] reported 100 and 53 known miRNAs in colostrum and mature milk using microarray, respectively. Ever since then, there have been various research reports on the expression of miRNAs in different milk fractions and across varying physiological conditions, highlighting their potential function in important biological processes such as milk production. For example, Li et al. [93] analyzed miRNAs in milk fat, whey, cells, and MG tissues and found 210, 200, 249, and 321 known and 33, 31, 36, and 176 novel miRNAs, respectively. Do et al. [94] also reported 475 known and 238 novel miRNAs among which 15 were significantly expressed across different lactation stages to regulate basic metabolic processes and cellular and immunological functions. For the bovine MG tissues, Li et al. [95] described 884 unique miRNA sequences (283 known, 505 novel, and 96 conserved miRNAs) (about 1100 miRNAs), while Le Guillou et al. [96] identified 167 novel miRNAs. Moreover, Luoreng et al. [97] identified 1838 miRNAs including 580 known miRNAs and 1258 predicted novel miRNAs in an experimental challenge of bovine MG with mastitis pathogens (*E. coli* or *S. aureus*). Li et al. [98,99] also reported 483 known bovine miRNAs and 139 novel miRNA hairpins in an experiment related to the heat stress response of bovine MG. Recently, Sun et al. [99] explored the expression profiles of miRNAs in 11 different tissues and indicated that the miR-1298, miR-2284b, and miR-376d are tissue-specific miRNAs for MG. Notably, many factors could influence miRNA expression profiles such as the sample collection procedures, breeds, as well as tools used for sequencing (RNAseq versus microarray), annotation, identification of novel miRNAs, etc. For instance, more than 1000 tools have been used to study, identify, or predict the targets of microRNAs (http://www.mirtoolsgallery.org/miRToolsGallery/, accessed on 25 August 2022). The choice of tools certainly affects the expression profiles of miRNAs. Recently, a new database called RumimiR (http://rumimir.sigenae.org/ accessed on 25 August 2022 [100]) was created for ruminant species and contains about 6808 unique microRNAs of bovine which should further improve bovine miRNA research.

### 3.3. Roles of miRNAs in Mammary Gland Infection and Mastitis

The healthy milk-producing MG that ensures the production of high-quality milk is essential to the dairy industry. Hence, a lot of resources are mostly invested in ensuring a mastitis-free dairy herd. As mentioned earlier, mastitis can be divided into clinical and subclinical types [101] and is caused by different pathogens [93] that can be diagnosed via somatic cell counting (SCC) and bacteriological culturing of milk samples. One general observation is that most studies of miRNA function in mastitis infection often focus on a particular pathogen challenge, except for a few studies that are performed by comparing the miRNA expression profile of positive and negative results from diagnostic methods. Regardless, there has been significant progress in identifying various dysregulated miRNAs in mastitis and understanding their contribution to the development and progression of the disease. Table 2 shows various differentially expressed miRNAs in mastitis while Table 3 lists the specific gene target of miRNAs for different mastitis pathogens and their function.

#### 3.3.1. *Escherichia coli*

*E. coli* remains one of the pathogens with the ability to cause severe systemic clinical mastitis symptoms. However, compared to the *S. uberis*, less attention has been paid to its resulting mastitis condition and that of *S. agalactiae* [67]. In brief, the severity of *E. coli* mastitis is mainly determined by host factors rather than by *E. coli* pathogenicity since host defense status is a key factor determining the outcome of the disease [67]. To understand the role of dysregulated miRNAs in this condition, Jin et al. [112] identified a total of 17 miRNAs that were DE between bovine mammary epithelial cells challenged with and without *E. coli*. Of these, five miRNAs (miR-184, miR-24-3p, miR-148, miR-486, and let-7a-5p) were specifically DE in *E. coli* infected cells. Interestingly, Ju et al. [111] also recently provided an insight into the relationship between bovine mastitis and the expression of miR-15a and miR-16a. Accordingly, miR-15a and miR-16a, which are mostly expressed and localized in the ductal and acinar cells of mammary gland tissues of the cows, were significantly upregulated in the mammary tissues and blood neutrophils of *E. coli*-infected cows, as compared to the healthy cows and contribute to the severity of the disease. The study further suggested that miR-15a/16a clusters may have a stronger regulatory effect on the target CD163 gene than each of them separately in mastitis cows. Additionally, Luoreng et al. [97] indicated that miR-200a, miR-205, miR-122, and miR-182 might be involved in immunity in the late stage of dairy cow mastitis caused by *E. coli*. All these studies confirm the significant role of miRNAs in the *E. coli*-related mastitis development.

#### 3.3.2. *Mycoplasma bovis*

A few studies have been devoted to understanding the roles of miRNAs on the *M. bovis*-caused mastitis. In an attempt to understand the immune response mechanism to mastitis infection, Özdemir [105] identified 24 known and 13 novel microRNAs that were differently expressed in *M.a bovis* positive tissues and demonstrated that let-7a-5p, let-7b, let-7c, let-7d, let-7e, let-7f, let-7g, let-7i, miR-1, miR-100, miR-101, miR-103, miR-106a, miR-106b, miR-107, and miR-10a might be involved in inflammation signaling pathways in mastitis cases caused by *M. bovis*. In another experiment, the author [126] also reported that the expression levels of miR-21, miR-146a, miR-155, miR-222, miR-383, miR-200a, miR-205, miR-122, and miR-182 were significantly upregulated in *M. bovis*-positive milk and that inflammation-related miRNA expression levels in the two different dairy cows’ (Holstein and Doğu Anadolu Kırmızısı) milk studied were altered in the presence of mastitis. The study suggested that the identified miRNAs could be used as biomarkers of bovine mastitis caused by *M. bovis.*

#### 3.3.3. *Staphylococcus aureus*

Perhaps the roles of miRNAs in mastitis caused by *S. aureus* are most studied compared to other mastitis related pathogens. Among the first studies, Jin et al. [112] performed an expression profiling of miRNAs in BMEC challenged with and without heat-inactivated *S. aureus* at 0, 6, 12, 24, and 48 hr and identified several known miRNAs (miR-2339, miR-21-3p, miR-423-5p, miR-499, miR-92a, miR-193a-3p, miR-23a, miR-99b, miR-21-3p, miR-193a-3p, miR-365-3p, miR-30c, and miR-30b-5p) that were significantly DE by the *S. aureus* challenge. Interestingly, a slower initial response of miRNAs to *S. aureus* bacteria (most DE miRNA reported after 12 hr infection) was also observed, indicating a slow progression of mastitis caused by *S. aureus*. Furthermore, Li et al. [127] identified many miRNAs (77) that were substantially DE between the *S. aureus*-infected and non-infected MG. Notably, miR-223, miR-1246, and miR-142-5p were significantly upregulated and miR-1, miR-23a, miR-31, miR-23b-3p, miR-26a, and miR-145 were significantly downregulated. Using target gene enrichments, the authors indicated that these DE miRNAs might be related to the regulation of the endocytosis pathway and olfactory transduction pathways involved in cancer. Chenet al. [128] validated the function of 25 miRNAs and genes associated with NF-kB signaling pathway between healthy and mastitis cows and proposed that the NF-kB pathway is activated in mastitis individuals because of a decreased inhibition of miRNAs. The study suggested that miR-16 and miR-223 may be used as new markers for the dairy mastitis prognosis based on the change in the expression level between the two groups. Ma et al. [129] identified 37 DE miRNAs (22 known and 15 novels) in the exosomes of milk that were naturally infected with *S. aureus*, as compared to the control group. Of those, miR-378 and miR-185 were significantly upregulated. These two miRNAs targeted the VAT1L, DYRK1B, MLLT3, HP1BP3, NPR2, and PGM1 genes which have been reported to be associated with various human diseases. Similarly, Cai et al. [109] also detected 18 DE miRNAs (12 upregulated and 6 downregulated) in the exosome of milk from *S. aureus*-infected cows as compared to that of healthy ones and predicted that the target genes of these miRNAs were significantly enriched in various biological processes, cellular components, and molecular functions related to an immune system process, response to stimulus, and growth. Four of these DE-expressed miRNAs (1 upregulated and 3 downregulated) were novel miRNAs. These DE miRNAs, especially miR-223 and miR-142-5p, could be considered potential candidates for early detection of mastitis.

The damage caused to the MG cells and tissue can be further aggravated by the persistence of pathogen infections through the late stages of mastitis, resulting in the loss of structural integrity of the alveoli, tissue necrosis, and reduction in milk quality [130]. Ju et al. [108] recently revealed the miRNome of bovine MG tissues at the late stage of natural infection with *S. aureus*. The study detected 29 DE miRNAs that regulate immune-related target gene expression affecting various cell and tissue responses to mastitis. The authors also reported a significant downregulation of miR-26a, which enhanced the expression of its target *FGA* gene that is involved in host defense, inflammation, and tissue damage [108]. They noted that the miRNA variations observed in the MG of the mastitis-infected cows can be linked to some processes such as maintenance of immune and defense responses, cell proliferation and apoptosis, and tissue injury and healing during the late stage of infection [108]. On the other hand, a recent experiment that examined the differential miRNA expression in the peripheral blood of dairy cows in response to mastitis infection by S. *aureus* revealed that the quantity of differentially expressed miRNA in the blood increased with the extension of S. *aureus* infection time, implying that the response of blood miRNA to inflammation mainly occurs at the late stage of mastitis [117]. This information may be particularly useful when developing targeted therapy for the treatment of the disease.

#### 3.3.4. *Streptococcus agalactiae*

To better understand the pathogenicity and defense mechanisms that are activated during mastitis disease, Lewandowska-Sabat et al. [119] conducted an miRNA expression profiling of macrophages derived from bovine blood monocytes that were infected in vitro with two strains of *S. agalactiae* (ST103 and ST12). The authors reported 17 and 44 DE miRNAs in the macrophages infected with ST103 and ST12, respectively, in comparison to the control cultures. Accordingly, two bacterial strains significantly regulated miRNAs that were found to be involved in the alternative activation of macrophages. Moreover, the DE miRNA in the macrophages infected with both bacteria strains and their predicted target genes were significantly enriched in different immune-related pathways. The study further examined different key immune genes such as the transcript level of tumor necrosis factor-alpha (TNFα), interleukin-6 (IL-6), interleukin-8 (IL-8), interleukin-10 (IL-10), interleukin-1 beta (IL-1β), and transforming growth factor-beta 1 (TGFβ1) during the early phase of infection. Their results showed that all the six key immune genes, except TGFβ1 which was downregulated by ST12, were significantly upregulated by both strains of *S. agalactiae*. Two (miR-155 and miR-125b) of the three DE miRNAs that were unique to the ST12 infection are involved in the regulation of TNF production during mycobacterial infection and the ST12-induced miRNAs were associated with a stronger inflammatory response than the ST103 [119]. The authors finally suggested that the DE miRNAs could be used to develop a marker assay for the early diagnosis of subclinical infections such as bovine streptococcal mastitis. Moreover, Pu et al. [115] identified 35 DE miRNAs in bovine MG tissues with the *S. agalactiae*-type mastitis. Their result further showed that miR-223 and miR-26a were the most upregulated and downregulated miRNAs, among the DE miRNAs, respectively. The DE miRNAs might regulate several immune responses and signal transduction pathways such as the RIG-I-like receptor signaling pathway, cytosolic DNA sensing pathway, and Notch signal pathway [115].

#### 3.3.5. *Streptococcus uberis*

*S. uberis* is among the most prevalent mastitis-causing pathogens throughout Europe and North America [131]. Therefore, several studies have attempted to investigate the role of miRNAs related to this pathogen using in vivo experiments challenging bacterial pathogens in mammary tissues of cows [112,113,114]. Naeem et al. [113] performed this experiment in 14 selected miRNAs (miR-10a, -15b, -16a, -17, -21, -31, -145, -146a, -146b, -155, -181a, -205, -221, and -223) in bovine mammary epithelial cell (BMEC) function in tissue challenged with *S. uberis*. The authors observed a significant downregulation of miR-181a, 16, and 31, and upregulation of miR-223 in infected versus healthy tissues. Furthermore, downstream enrichment of target genes indicated potential roles of miR-181a on intra-mammary infections via its regulatory function on FcgammaR-mediated phagocytosis, toll-like receptor signaling, and antigen processing and presentation pathway [113]. Lawless et al. [114] profiled the expression of miRNAs in primary bovine mammary epithelial cells (BMECs) at 1, 2, 4, and 6 h post-infection with *S. uberis* and reported 21 miRNAs including 20 known miRNAs that were significantly differentially expressed (DE). The target genes of miRNAs, which were downregulated in *S. uberis*-infected BMECs, were also enriched for pathways related to innate immunity [114]. Ngo et al. [110] studied circulating miRNAs in the milk of mastitis cows with a ‘natural level of exposure’ and in response to various causative agents ‘on farm’. The authors identified 26 miRNAs as generic indicators of clinical mastitis, and of those, seven miRNAs (miR-27b, miR-152, miR-194, miR-200b, miR-222, miR-379, and miR-18397) were suggested as early mastitis indicators. Moreover, 27 miRNAs that are unique to *S. uberis*-positive mastitis were identified and miR-320a/b were emphasized to have important roles given their link to modulation of trained immune activity [110].

#### 3.3.6. CMT Tests and Other Mastitis Pathogens

**Figure 3:**
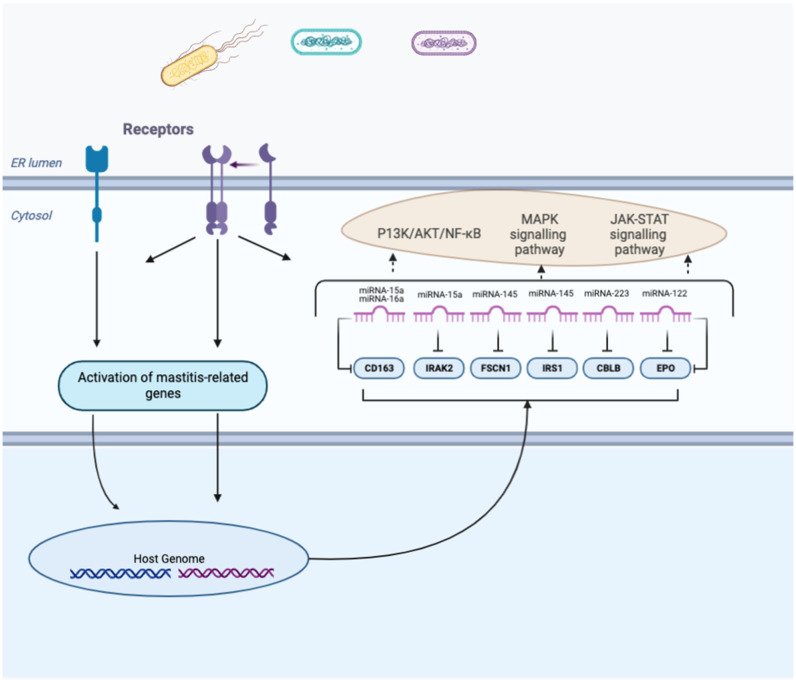
Schematic representation of confirmed miRNA–mRNA (gene) interaction in mastitis-induced tissues/cell. Infection by pathogens activates cascades of events that cause dysregulation of signification pathways, leading to inflammation of the udder. Each gene represented here is regulated by the respective miRNAs, subsequently dysregulating any of the represented signaling pathways (see Table 3).

Another interesting study for mastitis disease was undertaken by Lai et al. [104], who tested the sensitivity and specificity of several selected miRNAs for California mastitis test positive (CMT+) milk. The authors reported that miR-21, miR-146a, miR-155, miR-222, and miR-383 were significantly upregulated in CMT+ milk. Additionally, these five miRNAs showed high (more than 80%) sensitivity and specificity for CMT+ milk. They also tested the potential of using miR-92a, miR-375, and let-7g as housekeeping genes for mastitis disease and suggested that miR-92a might be a good candidate housekeeping gene for studies of miRNA function in mastitis diseases. Li et al. [132] reported that the relative expression of miR-144-5p and miR-130b-5p in mastitis-infected mammary tissues was significantly downregulated and upregulated by 3.34-fold, respectively, compared to healthy tissues, using stem-loop quantitative real-time polymerase chain reaction (RT-PCR). Similarly, using customized miRNAQTLsnp software, Jiang et al. [133] identified 5252 miRNA-related SNPs that could influence susceptibility to mastitis. They demonstrated that these SNPs are one of the plausible mechanisms underlying mastitis by modulating the interaction of miRNAs and immune-related genes such as the Spi-1 proto-oncogene (SPI1), which is a vital regulator of the innate and adaptive immune systems; and that the SNPs, including the confirmed rs109462250 SNP of miR-2899 (in the study), may have implications for the mastitis resistance breeding program in cattle. Further studies have shown the potential of using miRNAs as an effective diagnostic tool in the detection of bovine mastitis. For example, Srikok et al. [102] analyzed 8 candidate miRNAs from bovine milk and revealed 3 DE miRNAs (miR29b-2, miR146a, and miR155) that could serve as candidate miRNAs for determining infected/non-infected milk statuses of cows. Particularly, miR29b-2 seems to have promising qualities as a bovine mastitis biomarker, especially in cases where the mastitis status of a milk sample cannot be determined based on only CMT results. Another genome-wide bovine milk transcriptome analysis conducted by Lai et al. [103] identified 25 DE miRNAs (23 upregulated and 2 downregulated miRNAs) in CMT+ milk, as compared to normal milk. The authors identified 3 highly expressed novel miRNAs that were associated with bovine mastitis and relatively highly expressed in milk. One of the unique miRNAs (designated as ‘chr26_14097′ in the study) had been previously reported in different bovine specimens including milk-isolated monocytes from *S. uberis*-infected cows, Mycobacterium *bovis*-induced alveolar macrophages, and whole blood, and has been suggested to have immune regulatory functions. The study concluded that the DE miRNAs could be involved in the progression of human cancers, infections, and immune-related diseases [103]. Another example study showing the importance of miRNA in mastitis is a recent study by Tzelos et al. [134] which tested the possibility of using four inflammation-associated miRNAs (miR-26a, miR-142-5p, miR-146a, and miR-223) as diagnostic biomarkers of bovine mastitis. Interestingly, these miRNAs are also significantly dysregulated in many pathogen-induced mastitis studies reported in this current review. In their study, they found that the four miRNAs have a significant correlation with the mRNA levels of HP, TNF, CXCL8, and IL1B genes which are known inflammatory markers in milk cells. With a high accuracy of 100% sensitivity and more than 81% specificity, they concluded that the miR-223 and miR-142-5p levels could identify early inflammatory changes in individual quarter milk samples (CMT1) in the study. A pictorial representation of some confirmed miRNA-mRNA network in mastitis, together with their downstream target pathway, is shown in Figure 3.

## 4. LncRNAs in Mastitis Disease

LncRNAs are a diverse collection of ncRNAs with emerging regulatory roles in many biological processes in every branch of life [135,136,137,138]. LncRNA transcripts are >200 nucleotides in length and constitute the largest portion of the mammalian ncRNA transcriptome [135]. LncRNAs more closely resemble mRNAs than other classes of ncRNAs, especially in their biogenesis pathways and form. Most lncRNAs are transcribed by the activities of RNA polymerase II, have a 5′ terminal methylguanosine cap, and are often spliced and polyadenylated [136]. Some non-polyadenylated lncRNAs may arise through alternative pathways that are probably expressed from RNA polymerase III promoters [139,140] or arise during splicing and small nucleolar RNA production [141]. Furthermore, some lncRNAs are regulated in different ways at different stages of their biogenesis, maturation, and decay [138].

In cattle, approximately 23,000 lncRNAs transcripts have been reported (www.noncode.org/, accessed on 25 August 2022 [142]). The numbers of lncRNAs vary among tissues [127,143,144,145,146,147] and methods used to identify them from RNA sequencing data [148]. Among the first studies of lncRNAs in bovine MG, Koufariotis et al. [145] characterized the lncRNA repertoire across 18 bovine tissues including the MG and reported 9778 transcripts expressed in MG. Later, Tong et al. [149] identified 184 lncRNAs (intergenic) in the bovine MG including 36 lncRNAs co-located with 172 milk-related quantitative trait loci (QTL) and 1 lncRNA co-located within a mastitis QTL region. LncRNAs are also important for MG health as several lncRNAs (H19, XIST, or TUB) have been reported to relate to mastitis diseases [150,151,152]. In an in vivo study, Wang et al. [152] reported that the lncRNA-TUB plays a crucial role in the morphological shape, proliferation, migration, and β-casein secretion of mammary epithelial cells, and could also mediate *E. coli*-induced inflammatory factor secretion and *S. aureus* adhesion to epithelial cells. Similar to TUB, lncRNA H19 and XIST also play important roles in immune-related processes. The lncRNA H19 has been shown to modulate TGF-β1-induced epithelial to mesenchymal transition in BMEC through the PI3K/AKT signaling pathway [151], while the lncRNA XIST mediates the BMEC inflammatory response via the NF-κB/NLRP3 inflammasome pathway. Recently conducted studies have revealed that lncRNAs play a significant role in *M. bovis* infections [153,154,155]. Wang et al. [3] and Wang et al. [155] showed that lncRNAs interact with the coding genes in both a cis and a trans way to carry out their regulatory functions. With an ever-growing number of WTSS studies, there also arose a need for prioritizing the SNPs from varied datasets. The challenge in identifying and inferring the causal mutations related to clinical mastitis was emphasized in [156]. Meanwhile, by hypothetically studying if the lncRNA expression profile is specifically changed in *M. bovis*-infected bovine mammary tissues, Özdemir et al. [157] determined the LncRNA–mRNA co-expression network playing an important role against *M. bovis*.

## 5. Circular RNAs and Other ncRNAs in Mastitis

Circular RNAs represent a large class of ncRNAs that have recently emerged as regulators of gene expression [158,159]. They were discovered several decades ago when Hsu et al. [160] investigated the splicing machinery processor for making precursor mRNA into mature viral mRNAs. As sequence data continually increase, the emerging functions of circRNAs are being determined and have been reported in RNA transcription, splicing, turnover, and translation [159]. Many scholars had pointed out that circRNAs may play an important role in many diseases, especially cancer [161,162] and can escape exonucleic acid degrading enzymes as a result of their lack of a free 5′ or 3′ end, making them more stable than miRNAs or other linear counterparts [158]. Consequently, circRNAs are considered useful biomarker candidates for disease diagnosis and therapy monitoring [158]. However, little research has been undertaken to characterize the circRNAs in ruminant species. The roles of circRNAs in lactation were also reported in cattle and goats [129]. Recently, Wang et al. [163] identified 2059 circRNAs from bovine colostrum and mature milk exosomes. By enrichment analysis, the authors noted that these circRNA host genes were involved in the cytoplasm, endoplasmic reticulum, transport, and transcription factor. Moreover, the study also showed that the circRNA profile of colostrum or mature milk exosomes differed greatly, which could be important for the milk recipients [163]. In another recent study, Wang et al. [164] reported differentially expressed circRNAs in lipopolysaccharide-induced mastitis using bovine mammary epithelial cells (bMECs) and found two novel circRNAs (named novel_circ_0004830 and novel_circ_0003097 in the study) which may endogenously regulate bovine mastitis by binding to inflammation-related microRNAs such as the bta-miR-145 that has been reported to regulate S. aureus-induced mastitis in MAC-T cells by regulating the FSCN1 gene expression level [121]. Unfortunately, not many studies have been undertaken on the role of circRNAs in mastitis infection to date and more research is needed in this area.

PiRNAs are 24–30 nt in length and are transcribed from genome regions consisting of transposable elements and other repetitive elements [165]. They are also involved in RNA silencing and regulate gene expression [165,166,167]. In humans, they are also viewed as promising biomarkers for several diseases such as cancers [168] while in bovines, several piRNAs might also contribute to early embryo development [169,170]. Recently, Testroet et al. [171] profiled ncRNAs in exosomes from raw milk and identified 88 piRNAs whose functions remain unknown. The roles of piRNAs in mastitis infection are still unknown.

## 6. Perspectives of ncRNAs Studies in MG Health and Mastitis

**Figure 4:**
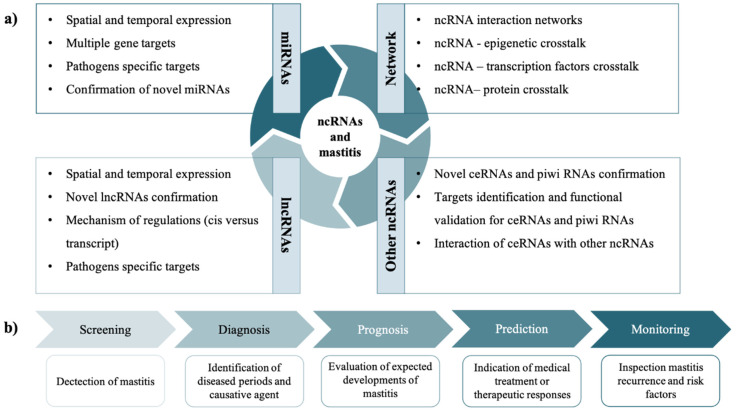
(**a**) lncRNA–miRNA–mRNA network in mastitis and (**b**) the different stages for the development of ncRNAs as biomarkers for mastitis infection.

Accumulated research has shown that ncRNAs play important roles in many processes related to MG health and mastitis. However, there is an existing gap in understanding the complete mechanisms of ncRNA regulation of mastitis developments. Constructions of the lncRNA–miRNA–mRNA network has the potential to highlight specific molecular functions and mechanisms and might be important for further exploration of ncRNAs in the disease (Figure 4a).

Previously, we also emphasized the importance of integrated omics approaches (genomics, transcriptomics, epigenomics, and proteomics) and wet-lab-based methods to identify and explore the potential roles of ncRNAs in mastitis disease [4]. The Functional Annotation of Farm Animal Genomes (FAANG) is involved in producing genome-wide data sets on RNA expression, DNA methylation, and chromatin modification, as well as chromatin accessibility and interactions and is helping to standardize the process of identifying ncRNAs and efficiently integrating the data. With no doubt, the FAANG is bringing hope of greatly improved genome sequences for domestic species.

Additionally, genome engineering is expected to significantly improve livestock production by precision genome editing [172,173,174], favoring markers associated with improved productivity, reproduction, and health status. In fact, genome editing technologies have been successfully used to edit the dairy cattle genome in a bid to ensure the production of specific milk types, such as the hypoallergenic milk (with less β-lactoglobulin protein) that causes fewer allergic problems, in dairy cows [175,176]. These tools have also been used to improve MG health by generating mastitis-resistant cattle [177,178]. Editing miRNA sequences has also helped to improve the milk components as evidenced in a transgenic calf that was engineered to express miRNA-4 and miR-6, which consequently showed an absence of β-lactoglobulin in its hormonally induced milk and a concurrent increase in casein proteins in the milk [179]. Although these technologies have great potential for future animal breeding, difficulties in large-scale application and integration in breeding schemes need to be overcome [180].

Furthermore, the development of a panel for early detection of bovine mastitis using ncRNAs is very crucial for tackling this infectious disease. However, the lack of specificity, especially among miRNAs that can respond to diverse infections, poses a challenge to this development. Therefore, there is a need to ensure that potential biomarkers have a high level of sensitivity and specificity or, alternatively, complimented with other disease-specific ncRNAs or miRNA to ensure diagnostic accuracy. Figure 4b shows the different stages involved in the development of ncRNAs as biomarkers that could aid the detection and treatment of mastitis disease. However, robust research would also be needed to confirm the pathological role of potential ncRNA biomarkers across breeds, in different environmental conditions, and at different times post-infection, as ncRNAs, especially miRNAs, have been shown to be differentially expressed in different mastitis conditions.

In addition, the development of high throughput phenotyping and sequencing, and the generation of big data provide great opportunities for dairy farms to cope with infectious diseases [181]; therefore, more comprehensive studies of ncRNAs on mastitis are expected to be performed based on larger and deeper phenotypes. For example, the International Dairy Data Exchange (iDDEN), a non-profit organization, has just been recently established to ensure efficient sharing of dairy cattle data among 13 different nations across the globe, and with structures like this in place, more data could be utilized for precise diagnosis of mastitis in dairy cows or development of better machine learning algorithms that can efficiently integrate generated phenotype and omics data for the improved detection methods for mastitis disease. Additionally, this data would be very useful in training different machine learning algorithms for early detection of mastitis syndrome, especially subclinical mastitis, even at a population or herd level, therefore reducing the use of antibiotics and the consequent risk associated with the emergence of antibiotic-resistant bacteria. Finally, the importance of high-performance computing and the machine learning approaches for pinpointing the roles of ncRNAs in each stage of mastitis disease development cannot be ignored.

In conclusion, in this review, we discussed the biology of mastitis, its prevalence across the globe, and its effect on dairy production. We examined the roles of ncRNAs in bovine mastitis based on relevant literature and noted ncRNAs (miRNA to be precise) that have been reported to play significant roles in the incidence of mastitis disease, even at different times post-infection. However, most of this information has not been well harnessed, especially in the development of a sustainable solution for the treatment or prevention of mastitis disease in dairy cows. We found that only a few studies have been undertaken regarding the roles of lncRNA, circRNA, and PiRNAs in bovine mastitis, and more work is needed in this area as these ncRNAs are potential biomarkers for different human diseases. We suggested that validated miRNAs could be used to develop a set of panels for the early detection, prognosis, and treatment of bovine mastitis. Although the practical application of the therapeutic approach to bovine mastitis might be quite costly for dairy cattle production, for now, we foresee a future possibility of developing efficient and cost-effective therapeutic drugs for the treatment of the disease.

## Figures and Tables

**Table 2:** Dysregulated miRNAs during mastitis infections.

Pathogens	Phenotypes/Tissues	Upregulated miRNAs	Downregulated miRNAs	References
California mastitis test positive (CMT+)	Milk	miR29b-2, miR146A, miR148s, miR155, and miR184.	miR24-2, miR181s1, and miR223	[102]
California mastitis test positive (CMT+)	Milk	miR-221, miR-146a, miR-10a, miR-142-3p, miR-223, miR-21-3p, miR-6529a, miR-338, miR-2284aa, miR-15a, miR-146b, miR-142-5p, miR-30f, miR-1246, miR-147, miR-2285b, miR-2285p, miR-222, miR-2284w, miR-132, miR-130b, miR-301a, miR-505	miR-23b-3p, miR-874	[103]
California mastitis test positive (CMT+)	Milk	miR-21, miR-122, miR-125b, miR-205, miR-222, and miR-383	miR-26b and miR-29b	[104]
*Mycoplasma bovis*	Milk	let-7a-5p, miR-100, miR-103, miR-107, miR-10a	miR-125a, miR-126-3p, miR-126-5p, miR-127, miR-1271	[105]
*Mycoplasma bovis*	Milk	miR-21, miR-146a, miR-155, miR-222, miR-383, miR-200a, miR-205, miR-122, and miR-182		[106]
*Staphylococcus aureus*	Milk	miR-1, miR-122, miR-1246, miR-146b, miR-142-5p, miR-146a, miR-154b, miR-184, miR-185, miR-196b, miR-205, miR-2340, miR-2889, miR-2904, miR-378, miR-378c, miR-451, and miR-378.	miR-218, miR-2320-3p, miR-369-3p, miR-582, miR-6525	[107]
*Staphylococcus aureus*	Milk	miR-1343-5p, miR-2407, miR-296, miR-2360, miR-2374, miR-2328-3p, miR-2412, miR-2904, miR-494, miR-2392, miR-2898	miR-2373, miR-423-3p, miR-126, miR-19b, miR-148a, miR-21, miR-31, miR-143, miR-26a, miR-145, miR-2881, miR-26b, miR-200b, miR-99a, miR-30a-5p	[108]
*Staphylococcus aureus*	Milk	miR-103, miR-142-3p, miR-142-5p, miR-146a, miR-146b, miR-147, miR-221, miR-223, miR-2284w, miR-2285b, miR-23a	let-7b, miR-1468, miR-423-5p	[109]
*Streptococcus uberis* *Coagulase Negative Staphylococcus*	Milk Milk	miR-1224, miR-2385-5p, miR-2433 miR-2344	miR-17-3p, miR-320a, miR-320b miR-1343-3p, miR-345-5p	[110]
*Escherichia coli*	Blood	miR-15a and miR-16a	-	[111]
*Escherichia coli*	MAC-T ^1^	miR-365-3p, miR-184 and miR-24-3p (6 hpi ^2^) miR-21-3p, miR-148a, miR-92a (12 hpi) miR-423-5p and miR-21-3p (24 hpi), miR-486 (48 hpi)	miR-193a-3p, miR-30c and miR-30b-5p (6 hpi) miR-423-5p (12 hpi) let-7a-5p, miR-184, miR-un5 miR-193a-3p (48 hpi)	[112]
*Streptococcus uberis*	BMEC ^3^	miR-223, mir-29e and mir-708 (2 hpi)	miR-181a, miR-16a, miR-31,	[113]
*Streptococcus uberis*	BMEC	let-7b, and miR-98 (4 hpi) miR-let-7c and miR-708 (4 hpi in normalized data) let-7b, miR-200c, miR-210, miR-24-2, miR-128-2, let-7d, miR-128-1, let-7e, miR-185, miR-652, miR-494, miR-2342 (6 hpi)	miR-29b-2, miR-193a, and miR-130a (4 hpi) miR-29b-2, miR-29c, miR-29e, and miR-100, miR-130a (6 hpi) miR-15a, miR-17, miR-26a-2, miR-29a, miR-29b-1, and miR-193a (in normalized data)	[114]
*Streptococcus aureus*	MAC-T	miR-2339 (6 hpi), miR-21-3p, miR-92a (12 hpi), miR-23a, miR-21-3p (24 hpi), miR-365-3p (48 hpi)	miR-423-5p and miR-499 (12 hpi) miR-193a-3p, miR-99b, miR-un5 (24 hpi) miR-193a-3p, miR-30c, and miR-30b-5p (48 hpi)	[112]
*S. agalactiae*	BMEC	miR-223, miR-2284k, miR-2484, miR-451, miR-383, miR-486, miR-2332, miR-122, miR-16a, miR-326	miR-26a, miR-33a, miR-335, miR-3660, miR-146a, miR-206, miR-628, miR-450b, miR-380-p, miR-1388-3p, miR-30e-5p, miR-23b-3p, miR-378b, miR-145, miR-136, miR-135a, miR-126-5p, miR-24, miR-4286, miR-450a, miR-3431, miR-2478, miR-23a, miR-487b, miR-331-5p	[115]
*Staphylococcus aureus*	Blood	miR-486, miR-451, miR-191, miR-342, and miR-30e-5p	miR-339b and miR-25	[116]
*Staphylococcus aureus*	Blood	miR-1301, miR-30b-5p, miR-193b, miR-320a, miR-19a, and miR-19b	miR-2284r, miR-144, miR-143, miR-205, and miR-24	[117]
*Escherichia coli*	Blood	miR-200a, miR-205, miR-345-5p, miR-671 (1 hpi) miR-545-3p, miR-190a, let-7a-3p, miR-345-5p, miR-592, miR-324, miR-411b, miR-153, miR-331-3p, miR-144, miR-2299-5p, miR-671, miR-32, miR-30b-5p, miR-29c, miR-1246, miR-142-3p, miR-29d-5p, miR-326, miR-27a-5p, miR-19a (3 hpi) miR-200a, miR-205, miR-182 (5 hpi) miR-200a, miR-205, miR-183, miR-214, miR-182, miR-199a-5p, miR-196a, miR-455-5p, miR-96, miR-143, miR-10b, miR-122, let-7a-3p, miR-126-5p, miR-144, miR-126-3p, miR-2285h, miR-345-5p, miR-3613a, miR-200c (7 hpi)	miR-122 (1 hpi) miR-122, miR-2450a, miR-193a-5p, miR-145, miR-200b, miR-2346 (3 hpi) miR-133a, miR-193b, miR-331-3p (5 hpi) miR-133a, miR-2332, miR-1388-3p, miR-342, miR-1291 (7 hpi)	[118]
*Streptococcus agalactiae* (ST12 and ST103 strain)	Blood	miR-221, miR-628, miR-146b, miR-2285m, miR-2284i, p-miR-3 (both strains) miR-425-5p, miR-425-3p, miR-30b-5p miR-223, miR-155, miR-500, miR-374b, miR-122 miR-2438 (ST12 strain) miR-708, miR-9-5p, miR-222, miR-7858 (ST103 strain)	miR-2427, miR-1306, miR-1249, miR-2898, miR-2478 (both strains) miR-2388-5p, miR-365-3p, miR-92b, miR-2431-3p, miR-197, miR-125a, miR-128, miR-328, miR-484, miR-1343-3p, miR-340, miR-30f, miR-30d, miR-125b, miR-505, miR-2284ab, miR-423-3p, miR-361, miR-92a, miR-1468 miR-669, miR-30c, miR-10a miR-2284w (ST12 strain) miR-2892, miR-1246 (ST103 strain)	[119]

^1^ Mammary alveolar cells; ^2^ hours post-infection; ^3^ bovine mammary epithelial cells; hpi: hour post infection.

**Table 3:** MicroRNAs with functionally validated target genes in mastitis infection using bovine mammary gland cells.

Pathogens	Phenotypes /Tissues	miRNAs	Target Genes	Main Consequences	References
*Escherichia coli*	Mammary tissues and blood neutrophils	miR-15a and miR-16a	*CD163*	Decreases *CD163* ability to induce the secretion of anti-inflammatory cytokines	[111]
*Staphylococcus aureus*	Mammary gland tissue	miR-15a	*IRAK2*	Might reduce the negative regulatory function of *IRAK2* and increase the apoptosis of mast cells	[120]
*Staphylococcus aureus*	Mac-T cells	miR-145	*FSCN1.*	Translationally repress *FSCN1* function; inhibit the proliferation of Mac-T cells, significantly reduce the secretion of IL-12 and TNF-α, and increase the secretion of IFN-γ	[121]
	BMEC	miR-145	*IRS1*	Post-transcriptionally regulate *IRS1* expression and decrease the proliferation of mammary epithelial cell through the MAPK signaling pathway	[122]
*Staphylococcus aureus*	Milk	miR-223	*CBLB*	Reduce LTA-stimulated inflammation in Mac-T cells by targeting *CBLB* and the PI3K/AKT/NF-κB downstream pathway	[123]
*Streptococcus agalactiae*	BMEC	miR-122	*EPO*	Regulates the JAK-STAT signaling pathway by downregulating EPO in the mammary gland	[124]
miR-375 knockdown disease condition	BMEC	miR-375	*NR4A1/ PTPN5*	NR4A1 is an important mediator in early inflammation that upregulates IκBα expression but inhibits NF-κB activation; PTPN5 negatively regulates the activity and localization of MAPK family members	[125]

## Data Availability

Not applicable.
